# An American View of State Aid for Hospitals

**Published:** 1910-11-26

**Authors:** Bailey B. Burritt

**Affiliations:** Assistant Secretary, State Charities Aid Association.


### SPECIAL INSTITUTIONAL ARTICLES.

### AN AMERICAN VIEW OF STATE AID FOR HOSPITALS.

By BAILEY B. BURRITT, Assistant Secretary, State Charities Aid Association.

(Paper read at the American Hospitals Association Conference.)

Within the brief limits of this paper it is my intention to emphasise three points:—

1. Voluntary contributors to the support of hospitals plus the number of patients paying for their care and treatment are relatively very few in number, and hospital care, so far as its support is not derived from public taxes, is, therefore, chiefly a burden on "the few."

2. The obligation of caring for the sick is more social and democratic than individual or oligarchic. (Throughout the paper I use this word with no suggestion of arbitrariness, but simply in its root idea of "the few.")

3. Adequate hospital provision, such as will most completely preserve and conserve the public health, can be best secured by a readjustment of our present system so as to throw the obligation of caring for the sick squarely upon "the many" rather than on "the few."

The report for Roosevelt Hospital, in the City of New York, mentions eight individual cash contributors for the year 1909. In addition there are fifty-eight endowed beds and also several large endowments. If we count all of the special endowments as annual contributors, because they yield annual interest, it is possible that the total annual number of voluntary contributors might be one hundred. Some $3,700 was contributed by the Hospital Saturday and Sunday Association, which, small though it is, represents a comparatively large number of contributors.

Now, the number of bed-patients admitted to Roosevelt Hospital during the same year was 3,791. The number of persons contributing, roughly 1,364, is but little more than one-third of this number.

Similarly at St. Luke's Hospital, in the City of New York, the number contributing is a little more than one-third of the number cared for.

Mount Sinai Hospital, in New York City, has some 5,000 annual contributors. It has also 152 endowed beds. Out of 6,997 persons during its last fiscal year, the number of pay-patients is 1,733. If we add this to the number of voluntary contributors, we find that approximately 6,733 made direct contributions towards the care and treatment of 6,997 sick people, a much larger proportion than at Roosevelt or St. Luke's. The number of contributors is almost as large as the number of patients, but still, as I maintain, is relatively few. Mount Sinai differs from Roosevelt or St. Luke's Hospital, however, in that it derives approximately one-eighth of its income from public taxation. About one-eighth of Mount Sinai is, therefore, supported by all the taxpaying individuals in the City of New York. Presbyterian Hospital, in New York, J. Hood Wright Hospital, and the Norwegian Hospital, of Brooklyn, tell the same tale.

We may conclude, then, from the foregoing that the burden of caring for the sick in our general hospitals, so far as they are not supported by public taxation, rests on a very few voluntary contributors, plus the percentage of paying patients; and that the two combined make, relatively speaking, only a very small total number of individuals. Now it is clear that so far as hospitals are supported by public taxation they are democratic or social, rather than oligarchic or individual in their method of support. All of the people in a given community contribute to the support of the hospitals in that community so far as their support is derived from public taxation. On the other hand, so far as hospitals are supported by pay-patients, by voluntary contributors, or by endowments, they rest on an individual or oligarchic basis of support. They are supported by "the few" rather than by "the many."

What is the extent to which these two methods of hospital support obtain at the present time? From the Census Report for 1904 on Benevolent Institutions, I learn that the ratio of public support of all general hospitals to private support, for the year 1903, was approximately two to five.

The care of the insane, feeble-minded, epileptic, etc., is in reality a hospital problem. Many of these were cared for in general hospitals before the development of our present special institutions. In considering the extent to which present hospital work of the country is supported by public taxation, or by society at large, some account should be made of this class of institutions. A special report of the Census Office on "The Insane and Feeble-minded," issued in 1904, shows that the total annual cost of caring for the insane, which is derived from public taxation exclusive of pay-patients, would make a total of nearly $30,000,000, as compared with $20,000,000 expended by private hospitals. This also leaves entirely out special institutions for the feeble-minded and epileptic, and hospitals maintained in connection with almshouses. These last, in some States, form a very important item.

I mention these facts simply to show that deriving the support of the care of the sick from public taxation is not a novel proposition. On the other hand, in the general hospital field alone about two-sevenths of the support of hospitals is derived from taxation, whereas if we include the treatment of all kinds of physical and mental diseases the amount of support derived from public taxation is probably at least twice as large as that derived from other sources. Moreover, I have neglected in this consideration the indirect factor of the effect of not taxing private hospitals. As pointed out repeatedly by Dr. Goldwater, this is in effect a partial support of the hospital by public taxation.

For the sake of clearness, let me recapitulate. In 1903 approximately two-sevenths of the support of general hospitals was social or democratic, whereas the remaining five-sevenths was individual or oligarchic. I now pass on to the second part of the paper, which attempts to demonstrate that the obligation of caring for the sick is more social than individual.

### Causes of Disease are more Social than Individual.

No matter what the disease may be, there are both individual and social factors in its cause. In some the individual factor seems to predominate, but in the majority the social factor is evidently the more important. By consulting the table of mortality from well-defined causes of death in the borough of Manhattan for the year 1908, I find that there were 2,808 deaths from the acutely contagious diseases, such as typhoid fever, malaria, smallpox, diphtheria, croup, and other epidemic diseases. These are pre-eminently social diseases, so far as their cause is concerned. We call them "epidemics," thus the very derivation of the word retains through the ages the thought that they are *social* afflictions. Two other acutely contagious diseases, gonorrhœa and syphilis, are known as "the social diseases." Before the ravages of this class of disease the individual alone, without the aid of society, is helpless. Society is the medium through which the disease is transmitted, and society is the agent which enforces isolation. Tuberculosis is another disease the cause of which is likewise pre-eminently social. If now we add the number of deaths from tuberculosis to the number of deaths from the acutely contagious diseases, we find that approximately one-fourth of all the deaths in the borough of Manhattan came from causes admittedly social.

In addition to this group of diseases there is another group in which the predominance of the social factor is only slightly less apparent. I refer to such diseases as pneumonia, puerperal septicæmia, bronchitis, meningitis, dysentery, etc. By adding the number of deaths from this group of diseases to the number from the first group, I find that, as in the borough of Manhattan for the year 1908, approximately two-thirds of all deaths are due to diseases the causes of which are essentially social. Even in the case of diseases in which the individual factor seems to be predominant an unsuspected social cause is frequently found. Take for example, cirrhosis of the liver, so frequently caused by the excessive use of alcohol; wherever lies the responsibility for the excessive use of alcohol, at least a significant portion of it is social. Many of the ætiological factors of Bright's disease, neurasthenia, organic heart disease, etc., are social. We might likewise claim that the causes of general intestinal diseases have a large social factor due to the lack of proper social regulations, with regard to pure food, etc. Accidents again are distinctly *social* causes. The point which I wish to make, and which, I think, is indisputable, is that in the larger majority of diseases which necessitate hospitals, the causes are *predominantly social.*

### Effects of Disease—Social more than Individual.

When we come to consider the effects of disease we are impressed on every side by the fact that they are *social.* However important it may be to cure a man from sickness, to relieve him from suffering, and free him from the bad effects of his sickness, it is vastly more important to cure him in order to prevent his being a source of infection to other members of society, and in order to preserve his social efficiency. The individual suffers from his disease, but the extent of the social effects from the disease do not end with the impaired social usefulness of the individual; it is handed on to subsequent generations, whether by heredity or by creating a more unfavourable environment for them. From whatever angle we consider the effects of disease, therefore, we are constrained to admit that they are primarily *social.*

### Prevention of Disease a Social Problem.

Strive as he may, the individual alone is helpless against disease. He must depend upon society for maintaining sanitary conditions which reduce the spread of disease. It is vastly more important to prevent disease than to cure it, and the burden of prevention rests primarily upon organised social activity. Several factors are essential in the prevention of disease:—

1. Complete knowledge of the causes and methods of treatment.
2. Complete socialisation of this knowledge, by making it active in the consciousness of individuals.
3. The establishment of social and police measures to secure sanitary conditions, and the provision of institutions for care and treatment of disease.

Each of these factors is essentially *social.* The first involves observation and collection of *social* data; the second involves the use of every *social* means of publicity, including the pulpit, the school, the Press; while the establishment of proper sanitary regulations involves organised activity through governmental agencies, boards of health, departments of charities, hospitals, etc. The fact that prevention of disease is primarily a *social* problem needs no proof.

### Care of the Sick a Social Problem.

If it is agreed that the causes of disease are *social*, that its effects are emphatically *social*, and that its prevention is almost wholly *social*, the care of the sick becomes obviously a *social* problem. The responsibilty of dealing with it rests upon "the many," and this cannot be thrown upon the shoulders of "the few" without a loss to both. "The few" cannot supply adequate facilities to conserve fully the health of all.

There is an individual element in this responsibility, and I do not propose to relieve the individual of it. I simply propose, by deriving the support of hospitals from taxation, that the responsibility of the few and the many would be distributed more evenly upon each.

To illustrate further the defects of the method of throwing the burden on "the few," take one typical instance. The five-year-old son of a resident of New York, drawing a meagre salary, contracted measles from one of the neighbours' children. It was a severe case, and the mother, unable to hire additional help, worked night and day to give the boy the care he needed. In spite of her utmost exertions the child grew worse, but in the end partially recovered. The mother finally broke down in caring for the child, and in a short time had well-developed signs of tuberculosis. The outcome was that the mother died, the child was never strong, and the father's economic efficiency was undermined at an early age through overstrain and worry. Under our unjust system the burden of these misfortunes was thrown upon individuals unable to carry it, although the original misfortune was caused by a disease distinctly social in origin, and although its effects were socially disastrous. This is by no means an extreme example, and it illustrates well the folly of an individualistic system of caring for the sick. Co-operation would have preserved the health of the child, the life of the mother, and the health and efficiency of the father. A distinctly social problem cannot be efficiently attacked until all the people shoulder the responsibility for its support. I do not see how we can secure the co-operation of the people in the support of hospitals unless we derive their support chiefly from taxation.

Such a proposition will elicit many strong objections—first, that the tax-payer is already overburdened. But the force of this objection will, I think, be removed when the whole of my idea is grasped. As long as publicly maintained and controlled hospitals are for the indigent sick only, as long as the taxpayer recognises that he has no right to treatment in the institutions unless he and his family fall in the pauper class, he will support them more or less grudgingly. But give him the same right to take advantage for himself and his family that he has in the case of the public school and his objection to supporting hospitals will disappear. If by contributing a little each year he knows that he is making it possible for himself and his family to be adequately cared for in time of sickness, the dread of sickness will disappear, and he will cheerfully contribute. I have already shown that he is now supporting two-sevenths of all hospital care and treatment, although securing no rights or privileges for himself and his family.

Another objection will be urged that it is not the function of Government, either municipal, county, or State, to maintain hospitals. I believe that it is quite as essential for the State to see to it that its citizens have strong, physical bodies, as it is for the State to undertake that its children are properly educated. Indeed, without the sound, physical body, the State might almost as well not educate its children. It is, therefore, entirely within the proper functions of Government to assume the responsibility of providing institutions for the care and treatment of sick persons. I see no way to attain this much-desired goal except to look squarely to public taxation as the source of revenue for hospital work.

### Method of Control of Social Care of the Sick.

If it is granted that it is appropriate to raise money for the care of the sick by public taxation, what is the more expedient method of controlling the expenditure of money so raised. There are at present two methods found side by side, each quite extensive in its application. The first is the method of subsidising private hospitals. The second is the method of establishing publicly maintained and controlled hospitals. But in one case, as I wish to emphasise, the control is directly responsible to all the citzens, whereas in the other case the control is so remote that the average citizen has little or no method by which to influence that control. At most, the control of "the many" is the control of a supervisory department of the Government, which may say that the incorporation must maintain such and such a standard, or cease to receive public money. It is entirely negative and restrictive. The positive element of developing the institution along popular lines is entirely wanting. I would urge the second or democratic method of control of hospitals' expenditures raised by public taxation, because it makes the control more directly responsive to the wishes of the people, and because I believe thoroughly in the average man and in the wisdom of giving him all the powers that he will assume. Those who temperamentally distrust the average man will for the same reason incline to the other method.

By some it is urged that turning over the control of the money to "the few" removes the work of hospitals from practical politics. There is, however, no more reason why there should be objectionable politics in connection with public hospitals than there is in connection with public schools. So far as my observation goes there is quite as much politics in the method involved of subsidising private hospitals from taxation as there is in establishing and maintaining public hospitals, pure and simple. I am even inclined to think that the type of politics involved in the former method is even more secretive, insidious, and, I might add, invidious, than the type which we have to contend with in connection with the second method. Unfortunately we have no recent reliable statistics which give information relative to the extent to which the two methods are applied. Nevertheless, the report of the Census Bureau on Charitable Institutions for 1904 would seem to indicate that this country is far from being completely wedded to the method of subsidising private hospitals. Owing to the prevailing system of individualistic support, however, there has been a widespread tendency, whenever any municipality finds itself confronted with the problem of caring for the indigent sick, to subsidise some existing institution. There is an increasing tendency, however, for municipalities, when the number of sick is large enough to warrant a special institution, to establish it themselves. The great objection to this method has been that such institutions, being for the indigent sick only, do not inspire the active interest of people in their standards, and these accordingly fall behind. I do not believe in institutions for the indigent sick any more than I believe in public schools for the education of the indigent healthy. Hospitals for the *indigent* sick will never have first-class standards. When they cease to be hospitals for the *indigent sick*, and become hospitals for *sick persons*, we may expect the publicly maintained and controlled hospitals to have standards quite as efficient as the private incorporated hospital.

The Function of the Private Incorporated Hospital.

The function of a private incorporated hospital for the treatment of the sick is the same as that for a private school for the education of children: to meet the needs of those able to secure better advantages for their children than can be given to all in the public institutions. Such persons should not be deprived of the opportunity of securing better hospital accommodation and treatment at an increased cost to themselves. Public hospitals should cease to be *charitable* institutions, and should become, in the best sense of that word, popular institutions, in which all, as direct or indirect taxpayers, have the right to treatment. In the same way private hospitals should cease to be *charitable*, and become institutions maintained for the care of sick persons abundantly able to pay. The manifest tendency in this direction, I think, should be accelerated.

But, after all, the main question is not as to the respective fields of public and private hospitals. The most serious question is that of getting sufficient hospital facilities, owing to the greater congestion of the population. Under present conditions they are not cared for. It is really, then, a case of public care *versus* private neglect, or public care *versus* no care. More people are constantly going to hospitals, and yet but a small percentage of persons go who ought to go. It is for this reason that a rapid growth of the idea of supporting public hospitals from taxation is inevitable. The growth of county hospitals for the care of tuberculosis in our own State of New York is an indication of present tendencies. The way also has been opened in the same State for the rapid extension of public hospitals, by the enactment of a law during the last session of the legislature, Chapter 558, Laws of 1910, which gives authority to any city, village, or town to establish public general hospitals for the care of the sick, and provides a carefully thought out method of procedure.

The plan of making hospitals democratic in their support and control is somewhat radical. I do not fear that. The sole question is whether or not it is rational? If it is theoretically the most reasonable method, then it is ultimately the most practical. Only when *all of the people* realise that hospitals are their institutions, maintained and controlled *by them*, for their care and treatment in case of sickness, will hospitals be adequately supported; only then will there be real interest on the part of the people at large in hospital matters.

